# Use of gliptins reduces levels of SDF-1/CXCL12 in bullous pemphigoid and type 2 diabetes, but does not increase autoantibodies against BP180 in diabetic patients

**DOI:** 10.3389/fimmu.2022.942131

**Published:** 2022-07-25

**Authors:** Antti Nätynki, Päivi Leisti, Jussi Tuusa, Outi Varpuluoma, Laura Huilaja, Kentaro Izumi, Sanna-Kaisa Herukka, Olavi Ukkola, Juhani Junttila, Nina Kokkonen, Kaisa Tasanen

**Affiliations:** ^1^ Department of Dermatology, PEDEGO Research Unit, Medical Research Center Oulu, Oulu University Hospital and University of Oulu, Oulu, Finland; ^2^ Department of Dermatology, Hokkaido University Graduate School of Medicine, Sapporo, Japan; ^3^ Institute of Clinical Medicine - Neurology, University of Eastern Finland and Department of Neurology, Kuopio University Hospital, Kuopio, Finland; ^4^ Department of Internal Medicine, Research Unit of Internal Medicine, Medical Research Center Oulu, Oulu University Hospital and University of Oulu, Oulu, Finland

**Keywords:** bullous pemphigoid, autoimmunity, BP180, gliptins, DPP4, SDF-1 (CXCL12)

## Abstract

The use of dipeptidyl peptidase 4 (DPP4) inhibitors, (also known as gliptins), is associated with an increased risk of bullous pemphigoid (BP), an autoimmune blistering skin disease. To explore the mechanism behind gliptin-associated BP we investigated circulating autoantibodies against the major BP autoantigen BP180 in serum samples from patients with type 2 diabetes (T2D) with preceding gliptin medication (n = 136) or without (n = 136). Sitagliptin was the most frequently prescribed gliptin (125/136 patients). Using an ELISA assay, we showed that IgG autoantibodies against the immunodominant NC16A domain of BP180 were found in 5.9% of gliptin treated and in 6.6% of non-gliptin treated T2D patients. We found that 28% of gliptin treated patients had IgG autoantibodies recognizing the native full-length BP180 in ELISA, but among non-gliptin treated the seropositivity was even higher, at 32%. Further ELISA analysis of additional serum samples (n = 57) found no major changes in the seropositivity against BP180 during a follow-up period of about nine years. In immunoblotting, full-length BP180 was recognized by 71% of gliptin treated and 89% of non-gliptin treated T2D patients, but only by 46% of the age-and sex-matched controls. The chemokine stromal derived factor-1(SDF-1/CXCL12) is one of the major substrates of DPP4. Immunostainings showed that the expression of SDF-1 was markedly increased in the skin of BP patients, but not affected by prior gliptin treatment. We found that the use of gliptins decreased the serum level of SDF-1α in both BP and T2D patients. Our results indicate that the autoantibodies against the linear full-length BP180 are common in patients with T2D, but seropositivity is unaffected by the use of sitagliptin.

## Introduction

Bullous pemphigoid (BP) is the most common autoimmune subepidermal blistering skin disease primarily affecting elderly people. It is characterized by intense pruritus and tense bullae (1). The major target of BP IgG autoantibodies is BP180, a transmembrane hemidesmosomal protein of basal keratinocytes (2). Around 85% of BP autoantibodies target the juxtamembranous extracellular 16th non-collagenous (NC16A) domain of BP180, and levels of anti-NC16A IgG antibodies correlate with the severity of BP (1, 3).

Epidemiological data have convincingly demonstrated that the most evident risk factors for BP are certain neurological diseases (4–7) and the use of dipeptidyl peptidase 4 (DPP4) inhibitors (gliptins) (8–14). Gliptins are oral anti-diabetic drugs that are widely used to treat type 2 diabetes (T2D) (15), but the exact mechanism by which the use of gliptins increases the risk for BP is unknown (13). Furthermore, data about the clinical and immunological similarities and differences of regular BP (rBP) and gliptin-associated BP (BP+g) are scarce and partially conflicting (13). The target of gliptins, the DPP4 protein (also known as CD26), is ubiquitously expressed and has multiple functions in various cell types (16). The various effects of the inhibition of DPP4/CD26 activity include, for example, regulation of inflammatory cells such as T lymphocytes (17). In addition to BP, DPP4 and its inhibition have been linked to several other skin diseases including psoriasis, atopic dermatitis, T-cell lymphoma, hypertrophic scars, and sclerotic disorders (17).

One of the best characterized substrates of DPP4 is stromal cell-derived factor-1 (SDF‐1 or CXCL12), a lymphocyte and monocyte attracting chemokine (18). Alternative splicing generates several SDF-1 isoforms of which SDF-1α is the most common (19). DPP4 cleaves the first two amino (N)-terminal amino acids of SDF-1 and inhibits its binding to CXC chemokine receptor 4 (CXCR4) or atypical chemokine receptor 3 (ACKR3, previously named CXCR7) (18, 20–22). CXCR4 is ubiquitously expressed in most cells and the SDF‐1/CXCR4 axis participates in embryonic neural and vascular development and later maintains homeostasis in tissues e.g., during leukocyte trafficking and skin inflammation (18). In the skin the expression of SDF‐1 is increased during wound healing in fibroblasts and injured keratinocytes (23, 24). Moreover, the cutaneous expression of CXCR4 and its ligand SDF-1 are upregulated in patients with atopic dermatitis (25), psoriasis and keratinocyte-originating basal and squamous cell carcinomas (26).

We and others have previously found that patients with an elevated risk for developing BP i.e., those with neurological disorder such as Alzheimer’s disease, multiple sclerosis, and Parkinson’s disease, have an increased prevalence of BP180 autoantibodies (27–30). In the present study we investigate how the use of gliptins influences the rates of seropositivity for anti-BP180 IgG autoantibodies in patients with T2D. Given the major role of SDF‐1 as a DPP4 substrate and lymphocyte attractant, and its involvement in inflammatory skin diseases, we have also analyzed the amount of SDF-1 in serum and skin samples from patients with BP.

## Material and methods

### Patients

Patients with T2D treated with gliptin medication (T2D+g) and age- and sex-matched T2D patients who were not using gliptins were recruited from the ARTEMIS (31) and OPERA (32) study cohorts. Information concerning medication, skin diseases and cutaneous symptoms was collected from hospital records and patient questionnaires. BP serum and skin samples were collected in the Department of Dermatology, Oulu University Hospital at the time of diagnosis as described earlier (33). Patients using gliptins at the time of BP diagnosis were designated as BP+g. Age-matched healthy control sera were collected in Kuopio University Hospital from patients attending the hospital for knee replacement operations (30). Additional age-matched healthy control sera were obtained from the OPERA cohort and the Northern Finland Biobank Borealis, Oulu, Finland (https://www.oulu.fi/university/node/38474). All subjects gave written informed consent before the sample collection. The study was performed according to the principles of the Declaration of Helsinki. Ethics Committees of the Northern Ostrobothnia Hospital District and Kuopio University Hospital approved the collection of patient samples. All available patients were invited to attend a follow-up visit approximately 9 years after the date of baseline sample collection. ELISA assays were performed on samples obtained at study baseline, and again at the follow-up visits, but only baseline samples were analyzed by immunoblotting.

### ELISA assays

A commercial MESACUP BP180 ELISA kit (Medical and Biological Laboratories Co., Ltd., Nagoya, Japan) was used to measure serum BP180-NC16A IgG autoantibodies according to the manufacturer’s instructions. The cut-off value for positivity was set at ≥ 9 U/ml.

The methodology for the detection of full-length BP180 (FL-BP180) autoantibodies has been previously described (34). Briefly, 96 well plates (Nunc Maxisorp, Thermo Fisher Scientific, Inc., Waltham, MA, USA) were coated with 125 ng/well of mammalian cell expressed DDDDK-tagged BP180 protein. Plates were incubated with 1:100 serum dilutions. Absorbances were measured at 450 nm using a Victor2 1420 multilabel plate reader (Wallac, Turku, Finland). The cut-off value for positivity was determined by obtaining the maximal Youden index value from a ROC curve analysis using 16 known seropositive BP samples and 27 samples from age-matched healthy controls. SPSS software was used to determine a cut-off value of 1.950, with sensitivity of 0.938 and specificity of 0.889 (**Supplementary Table 1** and **Supplementary Figure 1**).

Serum SDF-1α levels were measured using a human CXCL12/SDF-1α Immunoassay (Quantikine^®^ ELISA DSA00, R&D Systems Inc., MN, USA) according to the manufacturer’s instructions.

### Immunoblotting and epitope mapping

FL-BP180 was expressed in COS7 cells transfected with human BP180 cDNA (35) and prepared as described previously (36). Fifty ng of each recombinant human glutathione-S-transferase (GST)-BP180-fusion protein expressed in *E. coli* spanning most of the BP180 polypeptide were used as an antigen. Immunoblotting and preparation of GST-BP180 fusion proteins were performed as described previously (30). Serum samples were diluted to 1:100 in 5% non-fat milk-TBS-0.1% Tween-20 and 1:50 000 peroxidase-conjugated anti-human IgG (Sigma-Aldrich, St. Louis, MO, USA) was used as a secondary antibody. Anti-GST (1:3000, Thermo Fisher Scientific, Rockford, IL, USA) with peroxidase-conjugated anti-rabbit IgG (Sigma-Aldrich) were used to detect fusion proteins. Protein bands were visualized with ECL Prime substrate (GE Healthcare, Buckinghamshire, UK) on a LAS Imager 3000 (Fujifilm, Tokyo, Japan). Epitope mapping data of 14 age and sex-matched healthy controls from our previous work (30) were used as a control.

### Immunohistochemistry

Three-µm thick sections of formalin-fixed and paraffin-embedded lesional skin samples of BP patients (n = 4 gliptin-treated, n = 7 without gliptin usage) and healthy control skin (n = 5) were deparaffinized and rehydrated. After heat-induced antigen retrieval in 10 mM Tris-1 mM EDTA (pH 9.0), the sections were immunostained with anti-human SDF-1α antibody (aa 1–93, 1:20000, PA5‐114344, Invitrogen/Thermo Fisher Scientific, Inc.) and a Rabbit-specific HRP/DAB (ABC) detection IHC kit (Abcam, Cambridge, UK). The average number of strongly stained infiltrated SDF-1 positive cells was calculated from the three areas with the most abundant positive cells in the epidermis and dermis using a x40 high power field objective. Stained cells in the blister fluid and inside capillaries were excluded from the analysis.

### Data analysis

Statistical analyses were conducted using the IBM SPSS software (v. 27) (IBM, Armonk, NY, USA). Differences between groups in FL-BP180 and BP180-NC16A levels (non-normally distributed, homoscedastic, symmetric distributions within groups, unequal group sizes) were analyzed using the Kruskal-Wallis test with Dunn’s corrected P values for multiple comparisons. Differences within the BP and T2D groups in SDF-1α measurements (non-normally distributed, heteroscedastic, unequal group sizes) were analyzed separately (2 families, 3 or 4 comparisons per family) using Welch’s ANOVA and Dunnett’s T3 adjustment for multiple comparisons. Mean and median values, 95% confidence intervals and percentiles were reported, as appropriate. The numbers of immunohistochemically stained SDF-1 positive cells in the skin samples were compared using the Kruskal-Wallis test with Dunn’s corrected P values for multiple comparisons. In epitope mapping, antibody-detected bands were densitometrically analyzed using the ImageJ software package (NIH, Bethesda, MD, USA) and classified in an ordinal scale: “0” = no band, “1” = weak, “2” = strong, “3” = very strong, as described previously (36). Differences between groups in epitope mapping were compared pairwise using the Fisher-Freeman-Halton exact test. A two-tailed P value of 0.05 or less was considered statistically significant.

## Results

### Study population

Our study included 136 T2D patients treated with gliptin medication (T2D+g) and 136 age- and sex-matched T2D patients who were not using gliptins from the ARTEMIS (31) and OPERA (32) study cohorts (**Table 1**). After an average interval of nine years after the baseline sampling (range 7–13 years), 179 patients were invited for the follow-up visit, 57 accepted the invitation and were subjected to serum sampling (**Table 1**). Total skin examination was performed to 28 patients who either had increased anti-BP180-NC16A values or reported skin symptoms in patient questionnaire.

**Table 1:** Characteristics and presence of BP180 IgG autoantibodies of T2D patients with (+g) or without use of gliptins at baseline and after a 9-year follow-up.

	T2D^†^	T2D+g^‡^	P value
n	136	136	
Females n (%)	47 (35)	48 (35)	ns.^§^
Age (mean ± SD^¶^ years)	66.7 ± 8.0	67.2 ± 7.8	ns.
BP180-NC16A-ab U/ml median (range)	2.0 (0.0–26.4)	2.3 (0.0–40.8)	ns.
BP180-NC16A-ab positives n (%)	9 (7)	8 (6)	ns.
FL-BP180-ab U/ml median (range)	0.9 (0.0–21.9)	0.1 (0.0–29.5)	ns.
FL-BP180-ab positives n (%)	44 (32)	38 (28)	ns.
	**Follow-up**	**Follow-up**
n	20	37	
Females n (%)	4 (20)	11 (30)	ns.
Age (mean ± SD years)	73.3 ± 6.7	73.0 ± 6.8	ns.
BP180-NC16A-ab U/ml median (range)	2.8 (1.5–12.2)	2.4 (1.0–18.8)	ns.
BP180-NC16A-ab positives n (%)	2 (10)	2 (5)	ns.
FL-BP180-ab U/ml median (range)	0.0 (0.0–2.0)	0.0 (0.0–90.3)	ns.
FL-BP180-ab positives n (%)	1 (5)	7 (19)	ns.

Follow-up cases were classified to T2D and T2D+g groups according to their use of gliptins at the time of follow-up, regardless of their use of gliptins at baseline. During the follow-up, 16 T2D cases started to use gliptins and were switched to the T2D+g group and 16 T2D+g cases stopped using gliptins and were switched to T2D group. Four T2D and 21 T2D+g cases remained in their respective groups from baseline to follow-up. IgG autoantibody levels in serum samples were measured using BP180-NC16A ELISA and full-length (FL) BP180 ELISA.

**^†^** Type 2 diabetes.

^‡^Type 2 diabetes with gliptins.

^§^Nonsignificant.

^¶^Standard deviation.

### Gliptin medication does not affect the prevalence of autoantibodies against folded BP180 in patients with type 2 diabetes

**Figure 1:**
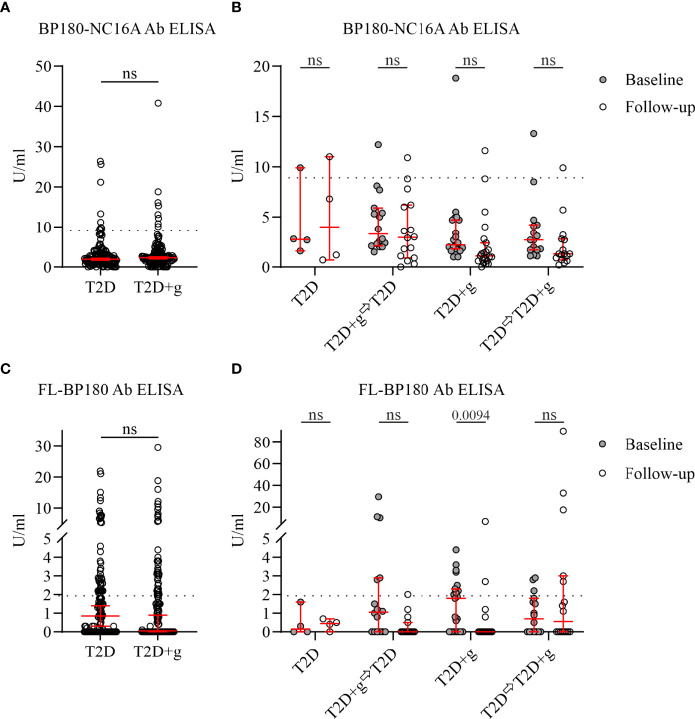
IgG autoantibodies against the NC16A domain of BP180 and full-length BP180 in serum samples of T2D patients with (+g) or without use of gliptins. Commercially available BP180-NC16A ELISA and full-length (FL) BP180 ELISA (in-house) with a cut-off value of 9.0 and 1.950 U/ml (dashed line), respectively, were used. n = 136 for both T2D and T2D+g groups at baseline **(A, C)**. In total, 57 follow-up serum samples were analyzed (B and D). **(A)** There was no significant difference in serum anti-BP180-NC16A IgG antibody levels between T2D and T2D+g groups at baseline. **(B)** Similar amounts of anti-BP180-NC16A IgG antibodies and numbers of positive cases were seen when the same individual cases were compared between baseline and follow-up. **(C)** Serum anti-FL-BP180 IgG antibody levels were similar in T2D and T2D+g groups at baseline. **(D)** Significant changes were seen between baseline and follow-up in anti-FL-BP180 IgG antibody levels in the T2D+g group: the number of positive cases decreased from 9 to 2 (one case was positive both in baseline and follow-up). Whiskers indicate 5th and 95th percentiles. Statistically significant differences between groups with associated two-sided P values are shown. ⇨, switch to; +g, gliptins; Ab, antibodies; BP, bullous pemphigoid; Ctrl, control; ns; nonsignificant; T2D, type 2 diabetes; U/ml, units per milliliter.

Although the exact details of the timing and duration of gliptin medication were not obtained in all cases, the information of type of gliptin used and other medications was available for all patients. At baseline 125 gliptin users had sitagliptin as the prescribed agent, eight had vildagliptin, two had linagliptin and one had saxagliptin. When serum samples were analyzed by ELISA, in the T2D+g group, 8 out of 136 sera (5.9%) were classified as positive for anti-BP180-NC16A IgG antibodies, as were 9 out of 136 (6.6%) in the non-gliptin T2D group (**Figure 1A** and **Table 1**).

The data collected for the 57 cases who participated to follow-up visit revealed that 21 of them had continued the use of gliptins from baseline to follow-up visit and four of them had not been using gliptin during the whole study period. Before the follow-up sampling 16 patients had stopped using gliptins and 16 patients who had not been receiving gliptins at baseline had started their use before the follow-up visit. At the follow-up sitagliptin was the prescribed agent for 36 patients and vildagliptin for one patient. Three patients who had BP180-NC16A ELISA values just above positive cut-off at baseline, remained weakly positive (9.9–11.6 U/ml) at follow-up. One gliptin user with a negative baseline value (8.8 U/ml) turned weakly positive (10.9 U/ml) although he/she had discontinued gliptin use during the follow-up period. Taken together, there were no major changes in anti-BP180-NC16A IgG positivity in either the T2D or T2D+g groups during the follow-up period (**Figures 1A, B** and **Table 1**).

All 272 baseline samples and 57 follow-up samples were further analyzed by ELISA for FL-BP180 seropositivity (**Figure 1C**). We found that 28% (38/136) of T2D+g sera recognized FL-BP180, but among the T2D cases the proportion of seropositive samples was higher, at 32% (44/136) (**Table 1**). Only 14% (8/57) of follow-up samples were positive (**Table 1**). In T2D patients who were gliptin users both at the baseline and follow-up, average level of IgG antibodies against FL-BP180 decreased from baseline to follow-up (**Figure 1D**). Also, the group that had discontinued gliptin usage before follow-up sampling had lower average anti-FL-BP180 antibody levels and fewer positive cases at follow-up than baseline, but the difference was not statistically significant (**Figure 1D**). In other groups, there were no clear changes in FL-BP180 antibody levels or positivity at the group level.

A diagnosis of BP was present in one T2D+g female patient at the age of 66 years (four years after the baseline, in 2012) who had initially been treated with sitagliptin but was changed to vildagliptin medication before BP onset. The diagnosis of BP was based on positive direct immunofluorescence findings, but the BP180-NC16A ELISA was negative. The patient’s BP180-NC16A ELISA value was 2.6 U/ml at baseline in 2008 and 7.8 U/ml at follow-up in 2020. Her FL‐BP180 ELISA was negative at both baseline and follow-up. No other patients who was positive for anti-BP180-NC16A or FL-BP180 autoantibodies had skin symptoms suggestive of BP.

### Use of gliptins does not induce major changes in anti-BP180 IgG autoantibody profiles in patients with type 2 diabetes

**Figure 2:**
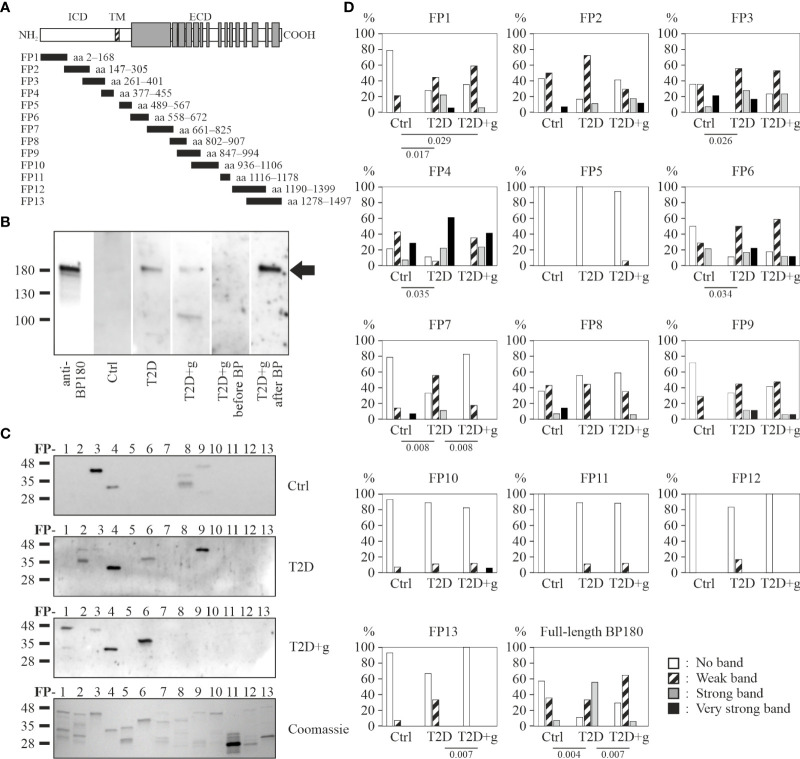
Detection of BP180 epitopes by IgG autoantibodies of T2D patients with (+g) or without use of gliptins and controls in immunoblotting. **(A)** Schematic representation of the full-length BP180 polypeptide and glutathione-S-transferase-BP180 fusion proteins. **(B)** Examples of recombinant FL-BP180 recognition by a specific BP180-antibody and serum of a healthy control, a T2D patient, a T2D+g patient and a T2D+g patient before and after BP diagnosis. The arrow indicates the BP180 band. **(C)** Fusion proteins were immunoblotted with 18 T2D, 17 T2D+g and 14 control serum samples. A representative Coomassie blue-stained gel of size-separated fusion proteins is also shown. **(D)** Relative frequencies of densitometrically classified immunoblotting signal intensities for indicated fusion proteins and statistically significant differences between groups with corresponding two-tailed P values are shown. Ctrl, control; ECD, extracellular domain; FP, fusion protein; ICD, intracellular domain; TM, transmembrane domain.

A subset of patient sera containing all BP180-NC16A ELISA positive (T2D+g: n = 8; T2D: n = 9), and ELISA negative baseline samples (T2D+g: n = 9, including the only BP T2D+g case; T2D: n = 9), and healthy controls (n = 13) were analyzed by immunoblotting against FL-BP180 (**Figures 2A, B**). We found that 89% T2D sera and 71% of T2D+g sera detected linear FL-BP180 in immunoblotting, while 46% of control sera contained anti-BP180 autoantibodies (**Table 2**). The recognition of FL‐BP180 in immunoblotting showed a weak tendency towards correlation with the FL-BP180 ELISA positivity in T2D group (**Table 2**).

**Table 2:** In immunoblotting full-length BP180 was weakly recognized by most T2D patient sera and at a lower frequency among T2D+g group.

	*FL-IB* ^‡^ *pos.*	*FL-IB neg.*	
T2D^†^ (n = 18)			
**NC16A-ELISA pos.** (n = 9)	***FL-IB pos.***	***FL-IB neg.***	Sum
FL-ELISA pos.	3 (33%)	0 (0%)	3 (33%)
FL-ELISA neg.	5 (56%)	1 (11%)	6 (67%)
Sum	8 (89%)	1 (11%)	9 (100%)
**NC16A-ELISA neg.** (n = 9)			Sum
FL-ELISA pos.	2 (22%)	0 (0%)	2 (22%)
FL-ELISA neg.	6 (67%)	1 (11%)	7 (78%)
Sum	8 (89%)	1 (11%)	9 (100%)
**Total** (n = 18)	**16 (89%)**	**2 (11%)**	**18 (100%)**
**T2D+g** ^§^ (n = 17)			
	***FL-IB pos.***	***FL-IB neg.***	
**NC16A-ELISA pos.** (n = 8)			Sum
FL-ELISA pos.	2 (25%)	1 (13%)	3 (38%)
FL-ELISA neg.	4 (50%)	1 (13%)	5 (63%)
Sum	6 (75%)	2 (25%)	8 (100%)
**NC16A-ELISA neg.** (n = 9)			Sum
FL-ELISA pos.	0 (0%)	1 (11%)	1 (11%)
FL-ELISA neg.	6 (67%)	2 (22%)	8 (89%)
Sum	6 (67%)	3 (33%)	9 (100%)
**Total** (n = 17)	**12 (71%)**	**5 (29%)**	**17 (100%)**
**Controls** (n = 13)			
	***FL-IB pos.***	***FL-IB neg.***	Sum
**Total** (n = 13)	**6 (46%)**	**7 (54%)**	**13 (100%)**

IgG autoantibodies targeting full-length BP180 in NC16A-ELISA positive and negative T2D and T2D+g sera and healthy control sera were analyzed by immunoblotting. IgG autoantibodies against native full-length BP180 and BP180-NC16A were measured by ELISA. The distribution of the (positive/negative) status in immunoblotting (FL-IB) and full-length BP180 ELISA (FL-ELISA) is shown.

**^†^** Type 2 diabetes.

^‡^Full-length BP180 immunoblotting.

^§^Type 2 diabetes + gliptins.

The same T2D+g, T2D and control serum samples whose anti-FL-BP180 autoantibodies were analyzed in immunoblotting were subjected to detailed epitope mapping using GST-fusion proteins (FP) covering the whole BP180 polypeptide (**Figure 2A**). Each serum detected a unique combination of several fusion proteins. When compared to previously published control serum results (30), T2D sera recognized intracellular and mid-extracellular epitopes slightly more frequently (**Figures 2C, D**). In addition, FP7 (aa 661–825) and FP13 (aa 1278–1497) were significantly more frequently recognized by T2D than T2D+g sera (**Figures 2C, D**, **Supplementary Tables 2–4**). Only one T2D+g patient had serum antibodies targeting the NC16A domain (FP5) in immunoblotting. Accordingly, the baseline and follow-up samples of this patient were positive in the BP180-NC16A ELISA, but no BP-related skin symptoms were found by the clinical examination.

### Gliptin treatment reduces the serum level of SDF-1α in patients with type 2 diabetes and bullous pemphigoid

**Figure 3:**
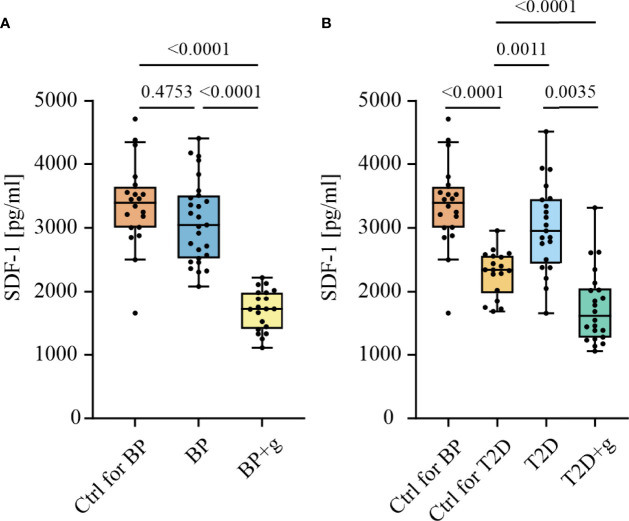
Concentrations of SDF-1α in serum samples of BP and T2D patients with (+g) or without use of gliptins. Serum SDF-1α levels were measured using human CXCL12/SDF-1α ELISA. **(A)** The use of gliptins was associated with decreased SDF-1α levels in BP patients (n = 20 for BP+g, 27 for BP and 20 for healthy age-matched control group [Ctrl for BP, 79.4 ± 2.1 years]). **(B)** The use of gliptins was associated with decreased SDF-1α levels in T2D patients (n = 22 for T2D+g, 21 for T2D and 18 for healthy age-matched control group [Ctrl for T2D, 62.7 ± 13.1 years]). Boxes depict the 25th and 75th percentiles (interquartile range) and line in the box is the median. Whiskers indicate 1.5 times the interquartile range. Statistically significant differences between groups with corresponding two-tailed P values are shown. +g, gliptin; BP, bullous pemphigoid; pg/ml, picograms per milliliter; T2D, type 2 diabetes; Ctrl, control.

Another approach to understand the mechanism behind gliptin-associated BP was to investigate how the use of gliptins in patients with BP and T2D modifies the amount of the chemokine SDF-1, one of the best characterized substrates of DPP4. We measured SDF-1α levels by ELISA in serum samples from patients with regular BP (rBP, n = 27, mean age ± SD = 81.6 ± 8.4 years), BP patients using gliptins (BP+g, n = 20, 77.7 ± 8.8 years) and age-matched healthy controls (n = 20, 79.4 ± 2.1 years). The level of SDF-1α of patients with rBP was similar to that found in controls (P = 0.4753) (**Figure 3A**). The serum level of SDF‐1α in BP+g patients was significantly lower than that in rBP patients (P <0.0001) and healthy controls (P <0.0001).

We also measured the amount of serum SDF-1α in the T2D+g (n = 22, 67.5 ± 9.6 years), T2D (n = 21, 64.5 ± 7.1 years), and age-matched healthy control groups (n=18, 62.7 ± 13.1 years). As was the case in BP patients, SDF-1α serum levels were significantly lower in T2D+g patients than in T2D patients (P = 0.0035). Compared to the healthy controls the level of SDF-1α was higher in patients with T2D (P = 0.0011) and lower in T2D+g patients (P <0.0001). SDF-1α levels of elderly BP controls were higher than in the younger T2D controls (P <0.0001) (**Figure 3B**). Serum SDF-1α levels did not correlate with BP180-NC16A-levels in either the BP or T2D patient groups (data not shown).

### The expression of SDF-1 is increased in the skin of patients with bullous pemphigoid

**Figure 4:**
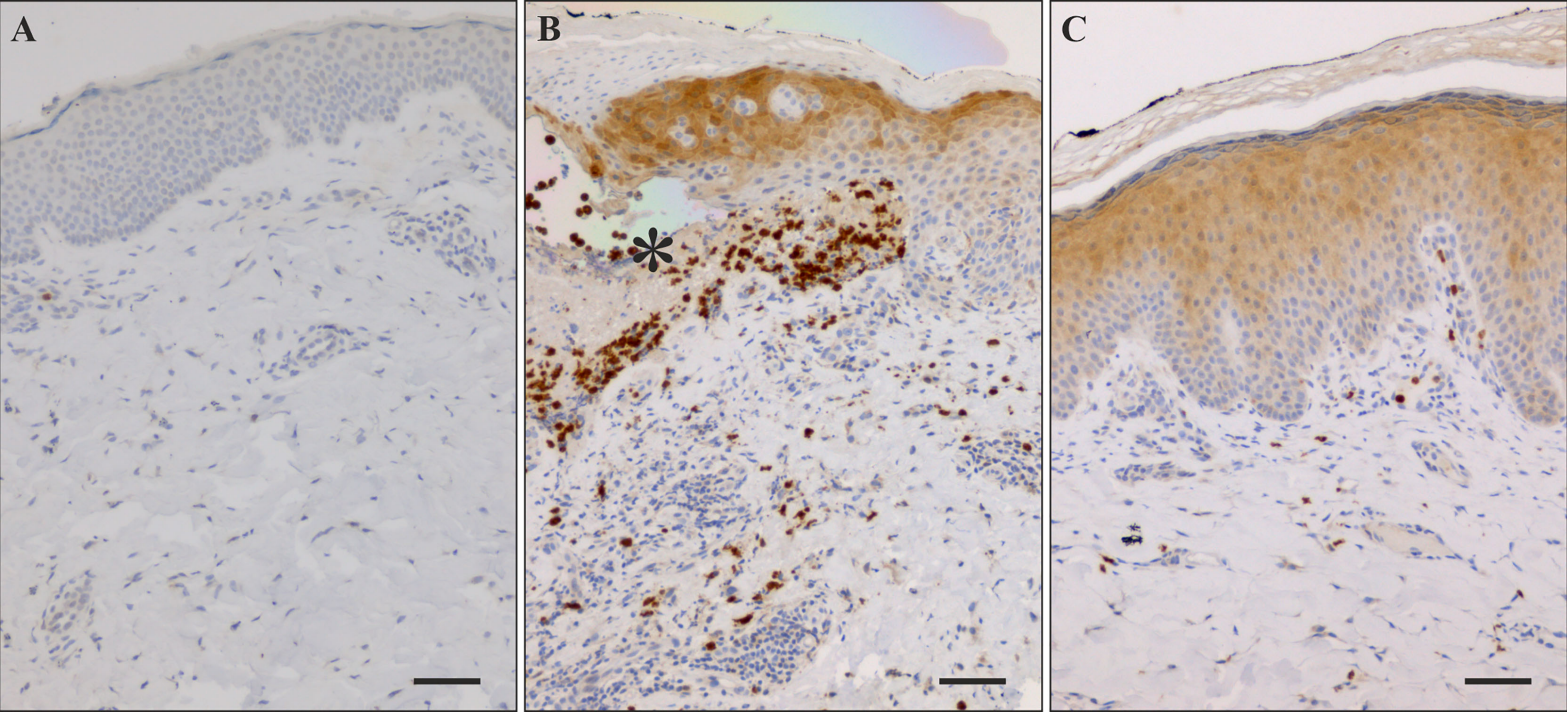
Immunostaining of SDF-1 in healthy control skin and lesional BP skin. **(A)** Immunohistochemical staining of SDF-1 was negative in healthy control skin. **(B)** Numerous SDF-1 positive cells were detected near blisters and in blister fluid (asterisk) in lesional BP skin along with strong staining of epidermis on the blister roof and on the margins of the blister. **(C)** In BP perilesional skin sections without blister there is strong staining of the upper layers of the epidermis and relatively few positive cells in the dermis. Scale bar = 50 µm.

Finally, we analyzed the expression of SDF-1 in the lesional rBP and BP+g skin, and healthy control skin. Immunostaining of the healthy skin was negative for SDF-1 in the epidermis and only a few immune cells showing strong staining were observed on the whole skin section (**Figure 4A**). Faint SDF-1 immunostaining was detected in capillary endothelial cells in both healthy control and BP skin. Independently of the gliptin status, strongly stained infiltrated cells were detected in the epidermis, dermis, and blister fluid in BP lesional skin (**Figure 4B**). SDF-1 was detected in epidermal keratinocytes, with the staining strongest at the blister roof and blister margins in the upper layers of the epidermis (**Figures 4B, C**). No statistical differences were found in the numbers of strongly stained immune cells between skin samples taken from patients with rBP and BP+g (data not shown).

## Discussion

In many autoimmune diseases, including type 1 diabetes, multiple sclerosis and rheumatoid arthritis, autoantibodies have been detected before clinical onset of symptoms (37). Accordingly, in a recent retrospective study of 18 BP patients, 22% were found to be seropositive for antibodies against BP180 and/or BP230, another BP-associated autoantigen, 1-5 months before the BP diagnosis. Interestingly, some cases had detectable autoantibodies over 10 years before the diagnosis (38). We and others have previously shown individuals who carry an elevated risk for developing BP due to Alzheimer’s disease or multiple sclerosis have IgG autoantibodies targeting the immunodominant NC16A domain and FL-BP180, despite the absence of clinical symptoms of BP (27–30). Although the risk of BP for patients being treated with gliptins for diabetes is at the same level or even higher than that of patients with neurological diseases (4, 14), studies concerning the preceding autoantibody levels before the diagnosis of gliptin-associated BP are scarce. Here, we investigated putative differences in the serological immunity against BP180 of patients with T2D. By using ELISA, we found that the prevalence of serum IgG-autoantibodies against folded BP180-NC16A or FL‐BP180 was similar among gliptin using and non-using T2D patients, but higher than we have previously found for healthy controls (7.5%) (30). Our results echo the findings of a Japanese study, which revealed no statistically significant difference in the prevalence of BP-autoantibodies based on gliptin treatment for T2D, although a tendency towards higher levels of anti-FL-BP180 autoantibodies was found in gliptin users (39).

In immunoblotting 71% of our patients with T2D+g had IgG autoantibodies against denatured FL‐BP180, but the proportion was even higher among T2D patients without gliptin medication, at 89%. Almost equal shares of BP180-NC16A ELISA positive and negative T2D+g and T2D samples recognized FL-BP180 in immunoblotting. Also 46% of the healthy control sera detected FL-BP180, which is in accordance with previous findings of our group and others (36, 40). Our previous immunoblotting works have shown that FL-BP180 is frequently recognized by serum samples from patients with Alzheimer’s disease, multiple sclerosis, dermatitis herpetiformis and coeliac disease. Of note is that those samples displayed various proportions of BP180-NC16A ELISA positive and negative sera (28, 30, 36). The high proportion of FL‐BP180 recognition in immunoblotting could be explained by the fact that the Alzheimer’s disease and T2D populations tend to be older than those with the other conditions. Indeed, elderly people have been shown to carry autoantibodies against BP180 but with low titer and low affinities (28, 40). This suggests the presence of “naturally occurring autoantibodies” against cryptic linear epitopes present in the FL‐BP180 (30). Resembling our previous results in patients with Alzheimer’s disease (30), the present study found a modest increase in the recognition of intracellular epitopes by T2D/T2D+g sera compared to controls. Although the T2D groups recognized the FP4 containing BP180 aa 377–455, the sera of T2D patients without gliptin usage had higher intensity bands than the T2D+g sera. Similarly, in our previous study of gliptin-associated BP, FP4 was more strongly detected by the non-gliptin-BP than gliptin-BP sera (33). Levels of autoantibodies against this linear epitope were also elevated in patients with multiple sclerosis (30). Together with the previously described gliptin-dependent recognition of FP4 by BP autoantibodies, the subtle differences between the epitope spectra of non-gliptin-T2D and gliptin-T2D samples suggest that gliptins might modify the degradation and/or antigen presentation of BP180.

The follow-up period of nine years should have revealed any incidence of BP or its autoantibodies amongst gliptin users, since the mean latency between the initiation of gliptin usage and the development of BP in different reports ranges from 3 to 27 months (8, 9, 12, 14, 33, 41). Although we were able to review the medical records of most participants, only 57 of the initial 179 attended the follow-up sampling, probably due to the Covid‐19 pandemic. During the study period sitagliptin has been the most prescribed gliptin in Finland (www.kela.fi/web/en/697), and consequently also in our T2D patients, sitagliptin was the most commonly prescribed gliptin. A study utilizing the EudraVigilance pharmacovigilance database revealed that vildagliptin was the gliptin most commonly reported to induce BP, having about 20‐fold higher proportional reporting ratio than sitagliptin (41). Several other studies, including Finnish population studies have similarly reported that the risk of BP is greater with vildagliptin than with sitagliptin (8, 14, 42). Because the majority of T2D patients in our study were using sitagliptin, we were not able to make any comparison between gliptins. However, an interesting detail is that the only T2D patient in our study population who developed BP during the follow-up period had initially used sitagliptin and developed BP after their medication was switched to vildagliptin.

To the best of our knowledge, the amount of SDF-1 in patients with BP has not previously been addressed. This is rather surprising considering the significant role of gliptins as a risk factor for BP, since SDF-1 is a well characterized substrate of DPP4 *in vivo* and is involved in inflammatory processes and lymphocyte chemoattraction (43). Because DPP4 inactivates many proinflammatory cytokines, inhibiting DPP4 with gliptins could be expected to lead to increased proinflammatory cytokine expression. Also, central B cell tolerance is dependent on the interaction of CXCR4 and SDF-1 as autoreactive B cells have a high expression of CXCR4 receptor, and SDF-1 signaling is required for the retainment of autoantibody-producing B cells in bone marrow (44). The blockade of CXCR4 in a mouse model elevated the number of autoreactive B cells in the spleen and blood (44). Gliptin-associated inactivation of SDF-1 could result in an increase of autoantibody-producing B cells in periphery. Finally, being a critical organizer of germinal centers during somatic hypermutation, changes in SDF-1 levels may also modify autoantibody production (45–47). In comparison to control samples, we detected stronger SDF‐1 staining in epidermal keratinocytes in BP lesional skin and in infiltrated inflammatory cells in the blister area but did not find any significant differences in SDF-1 immunostaining between rBP or BP+g skin. This is not parallel to decreased SDF-1α levels in BP+g serum but could be explained by the fact that ELISA assay detects specifically SDF-1α while the antibody used in immunohistochemistry recognizes all SDF-1 forms. In addition, it has been shown that the local paracrine expression of SDF-1 in tissues is not tightly bound to systemic levels in serum (48). Faint SDF‐1 positivity was found in vascular endothelial cells of both healthy control skin and BP lesional skin. It has previously been shown that in the healthy skin SDF-1 is mainly expressed in dermal fibroblasts, vascular endothelial cells, and epidermal dendritic cells (Langerhans cells) (26). The cutaneous expression of SDF‐1 is greater in the margins of healing wounds (23, 24, 49, 50) and in skin diseases such as atopic dermatitis, psoriasis and keratinocyte cancers (25, 26). DPP4/CD26 is highly expressed on the surface of T cells, and it may have a role in T cell differentiation, maturation, and proliferation (51). Gliptins could affect T cell migration by activating/inhibiting chemokines that are DPP4 substrates. For instance, the N-terminal amino acids of SDF-1 are required for binding to CXCR4 and therefore cleavage of the N-terminus by DPP4 blocks the chemotactic effect on lymphocytes (reviewed in 18). Sitagliptin treatment has been shown to change the T cell subpopulations of T2D patients, particularly lowering numbers of Th17 and regulatory T cells (Tregs) (52, 53). Tregs maintain immune tolerance by suppressing T cell activation (54). Dysfunction of Tregs was shown to induce BP-autoantibody production (55, 56) and depletion of Tregs induced inflammation and blistering in a mouse model of BP (57). However, a recent Japanese study found no significant difference in the counts and subpopulations of circulating Tregs between BP+g patients and controls while numbers of Tregs were increased in rBP (58). It remains to be clarified whether gliptins may enhance BP development in T2D patients’ skin *via* an imbalance in lymphocyte subpopulations and BP180-specific Tregs.

Our results confirmed the previous findings that serum SDF-1α levels are high in the elderly (59, 60) and in patients with T2D (61). Despite the increased expression in the BP skin, the level of SDF-1α in our patients with rBP was similar to those of the age-matched controls. However, we found that the levels of circulating SDF-1α were lower in BP and T2D patients using gliptins than in those without gliptin treatment. Of note is that the ELISA assay we used does not discriminate between the full-length (aa 1–68) and DPP4 trimmed (aa 3–68) SDF-1α. In line with our results, the use of gliptins for three months has been shown to decrease circulating total SDF-1α levels in T2D patients (62, 63). In a more specific analysis circulating levels of intact SDF-1α increased while levels of truncated (DPP4-cleaved) SDF-1α decreased both in T2D patients using sitagliptin (64, 65) and in animals with gliptin administration (66). Platelets are a major source and site of storage of SDF-1 (67) and recently many proteins associated with platelet degranulation have been detected in BP blister fluid (68). Also, elevated serum levels of sP-selectin in BP patients reflect platelet activation (69). T2D patients have enhanced platelet activation and increased risk for atherosclerosis (70). Gliptins may regulate platelet functions; sitagliptin has been shown to reduce platelet aggregation (71, 72). Taken together, high serum levels of SDF-1α could be a sign of platelet activation in both BP and T2D patients and in elderly controls (73), but it seems that the decline of circulating SDF-1α is a BP-independent gliptin-induced phenomenon. One possible explanation for gliptin-induced decline of SDF-1α is the ACKR3-mediated scavenging of SDF-1 during prolonged use of gliptins (48, 74).

Currently we have limited knowledge concerning the mechanism and timing of progression from a preclinical state with BP autoantibodies to clinical disease and whether these vary in BP patients with different risk factors. Here we found that the use of a gliptins, more specifically sitagliptin, does not induce significant appearance of anti-BP180 autoantibodies but modulates levels of SDF-1α decreasing its total serum concentration in both T2D and BP patients. Considering the limitations caused by small study populations, it seems that seropositivity against BP180 is more common among patients with neurological risk factors than gliptin users. Although rather challenging to implement, we need further research using serum samples obtained at various time points well-ahead of the onset of BP symptoms from a large number of patients with various risk factors.

## Data Availability Statement

The original contributions presented in the study are included in the article/**Supplementary Material**. Further inquiries can be directed to the corresponding author.

## Ethics Statement

The studies involving human participants were reviewed and approved by Ethics Committee of the Northern Ostrobothnia Hospital District and Ethics Committee of the Kuopio University Hospital. The patients/participants provided their written informed consent to participate in this study.

## Author Contributions

Conceptualization: JT, NK, KT. Methodology: AN, PL, JT, OV, LH, KI, S-KH, OU, JJ, NK, KT. Analysis: AN, JT, PL, KT. Supervision: JT, NK, KT. Writing: – original draft preparation: AN, JT, NK, KT. Writing – Review and editing: AN, PL, JT, OV, LH, KI, S-KH, OU, JJ, NK, KT. All authors read and approved the final manuscript.

## Funding

This study was supported by research grants from the Academy of Finland, the Sigrid Juselius Foundation, the University of Oulu Graduate School and the Medical Research Center, Oulu University Hospital (MRC Oulu).

## Acknowledgments

Ms Anja Mattila, Ms Päivi Kastell, Ms Saija Kortetjärvi, Ms Ms Erja Tomperi and Ms Leena Ukkola are acknowledged for their excellent technical assistance.

## Conflict of Interest

The authors declare that the research was conducted in the absence of any commercial or financial relationships that could be construed as a potential conflict of interest.

## Publisher’s Note

All claims expressed in this article are solely those of the authors and do not necessarily represent those of their affiliated organizations, or those of the publisher, the editors and the reviewers. Any product that may be evaluated in this article, or claim that may be made by its manufacturer, is not guaranteed or endorsed by the publisher.
