# Supplementary material for: Use of gliptins reduces levels of SDF-1/CXCL12 in bullous pemphigoid and type 2 diabetes, but does not increase autoantibodies against BP180 in diabetic patients
Source: Front Immunol. 2022 Jul 25;13:942131. doi: 10.3389/fimmu.2022.942131 (PMC9357937; doi:10.3389/fimmu.2022.942131)

Supplementary Material

# Supplementary Tables

**Supplementary Table 1. Determination of the cut-off value for full-length BP180 IgG antibody ELISA using a received operator characteristic (ROC) curve.** Sixteen bullous pemphigoid (BP) sera and 27 healthy control sera were used to determine the cut-off value for full-length BP180 ELISA positivity yielding optimal sensitivity and specificity. Full-length BP180 ELISA index values of each serum sample were input into SPSS statistical software and an ROC analysis was performed. From the analysis output, the Youden index value was calculated by subtracting 1 minus specificity from sensitivity. The maximum Youden index value and corresponding cut-off value are highlighted in bold. Sensitivity and specificity were calculated using the cut-off value of 1.950 U/ml from known positive and negative samples as sensitivity = true positive / (true positive + false negative) and specificity = true negative / (true negative + false positive).

| Cut-off values of FL-BP180 ELISA^†^ | Sensitivity | 1 - Specificity | Youden-index |
| --- | --- | --- | --- |
| -1.00 | 1.00 | 1.00 | 0.000 |
| 0.05 | 0.938 | 0.556 | 0.382 |
| 0.50 | 0.938 | 0.519 | 0.419 |
| 0.95 | 0.938 | 0.481 | 0.456 |
| 1.05 | 0.938 | 0.407 | 0.530 |
| 1.25 | 0.938 | 0.296 | 0.641 |
| 1.55 | 0.938 | 0.259 | 0.678 |
| 1.75 | 0.938 | 0.222 | 0.715 |
| 1.85 | 0.938 | 0.148 | 0.789 |
| **1.95** | **0.938** | **0.111** | **0.826** |
| 2.10 | 0.875 | 0.111 | 0.764 |
| 2.25 | 0.813 | 0.074 | 0.738 |
| 2.75 | 0.750 | 0.074 | 0.676 |
| 4.00 | 0.750 | 0.037 | 0.713 |
| 4.95 | 0.750 | 0.000 | 0.750 |
| 7.95 | 0.688 | 0.000 | 0.688 |
| 12.85 | 0.563 | 0.000 | 0.563 |
| 16.70 | 0.500 | 0.000 | 0.500 |
| 20.35 | 0.438 | 0.000 | 0.438 |
| 31.40 | 0.375 | 0.000 | 0.375 |
| 51.10 | 0.313 | 0.000 | 0.313 |
| 76.55 | 0.250 | 0.000 | 0.250 |
| 95.75 | 0.188 | 0.000 | 0.188 |
| 124.20 | 0.125 | 0.000 | 0.125 |
| 221.80 | 0.063 | 0.000 | 0.063 |
| 296.20 | 0.000 | 0.000 | 0.000 |
|  |  |  |  |

^†^The smallest cut-off value is the minimum observed test value minus 1, and the largest cut-off value is the maximum observed test value plus 1. All the other cut-off values are the averages of two consecutive ordered observed test values.

**Supplementary Table 2. Frequencies of fusion proteins recognized by sera of type 2 diabetes patients with or without use of gliptins and healthy controls in immunoblotting.** BP180 epitope mapping was performed by immunoblotting. A total of 13 BP180-GST fusion proteins (FP1–FP13) and full-length recombinant human BP180 (BP180) detected by serum IgG antibodies of type 2 diabetes patients without gliptin medication (T2D) or with gliptin medication (T2D+g) and healthy controls (Ctrl) were densitometrically quantified and classified into four categories: 0 = no band, 1 = weak band, 2 = strong band, 3 = saturated/very strong band. Ctrl group data concerning FP recognition was obtained from our previous work (Tuusa et al., 2019).

|  | **Inte-nsity** | | **FP** | | | | | | | | | | | | | **BP180** |
| --- | --- | --- | --- | --- | --- | --- | --- | --- | --- | --- | --- | --- | --- | --- | --- | --- |
|  |  |  | **1** | **2** | **3** | **4** | **5** | **6** | **7** | **8** | **9** | **10** | **11** | **12** | **13** |  |
| Ctrl | | 0 | 11 | 6 | 5 | 3 | 14 | 7 | 11 | 5 | 10 | 13 | 14 | 14 | 13 | 7 |
|  |  | 1 | 3 | 7 | 5 | 6 | 0 | 4 | 2 | 6 | 4 | 1 | 0 | 0 | 1 | 4 |
|  |  | 2 | 0 | 0 | 1 | 1 | 0 | 3 | 0 | 1 | 0 | 0 | 0 | 0 | 0 | 2 |
|  |  | 3 | 0 | 1 | 3 | 4 | 0 | 0 | 1 | 2 | 0 | 0 | 0 | 0 | 0 | 0 |
|  |  | Total | 14 | 14 | 14 | 14 | 14 | 14 | 14 | 14 | 14 | 14 | 14 | 14 | 14 | 13 |
| T2D | | 0 | 5 | 3 | 0 | 2 | 18 | 2 | 6 | 10 | 6 | 16 | 16 | 15 | 12 | 2 |
|  |  | 1 | 8 | 13 | 10 | 1 | 0 | 9 | 10 | 8 | 8 | 2 | 2 | 3 | 6 | 6 |
|  |  | 2 | 4 | 2 | 5 | 4 | 0 | 3 | 2 | 0 | 2 | 0 | 0 | 0 | 0 | 10 |
|  |  | 3 | 1 | 0 | 3 | 11 | 0 | 4 | 0 | 0 | 2 | 0 | 0 | 0 | 0 | 0 |
|  |  | Total | 18 | 18 | 18 | 18 | 18 | 18 | 18 | 18 | 18 | 18 | 18 | 18 | 18 | 18 |
| T2D+g | | 0 | 6 | 7 | 4 | 0 | 16 | 3 | 14 | 10 | 7 | 14 | 15 | 17 | 17 | 5 |
|  |  | 1 | 10 | 5 | 9 | 6 | 1 | 10 | 3 | 6 | 8 | 2 | 2 | 0 | 0 | 11 |
|  |  | 2 | 1 | 3 | 4 | 4 | 0 | 2 | 0 | 1 | 1 | 0 | 0 | 0 | 0 | 1 |
|  |  | 3 | 0 | 2 | 0 | 7 | 0 | 2 | 0 | 0 | 1 | 1 | 0 | 0 | 0 | 0 |
|  |  | Total | 17 | 17 | 17 | 17 | 17 | 17 | 17 | 17 | 17 | 17 | 17 | 17 | 17 | 17 |

**Supplementary Table 3. IgG autoantibodies in the sera of type 2 diabetes patients with or without use of gliptins and controls recognize different BP180 fusion proteins in immunoblotting.** Fusion proteins (FP) with significantly inhomogeneous recognition between T2D patients with or without use of gliptins and control group are shown. The significance threshold was set at 0.05. P values below this and their corresponding test values are highlighted in bold.

| **Fusion protein** | **Fisher-Freeman- Halton's exact test** | **Exact 2-sided P value** |
| --- | --- | --- |
| FP1 | **11.971** | **0.024** |
| FP2 | 9.612 | 0.088 |
| FP3 | **12.580** | **0.034** |
| FP4 | **11.902** | **0.044** |
| FP5 | 1.796 | 0.633 |
| FP6 | 9.589 | 0.121 |
| FP7 | **13.714** | **0.006** |
| FP8 | 5.895 | 0.353 |
| FP9 | 5.899 | 0.396 |
| FP10 | 2.226 | 0.944 |
| FP11 | 1.676 | 0.535 |
| FP12 | 3.800 | 0.101 |
| FP13 | **7.655** | **0.009** |
| BP180 | **16.840** | **0.001** |

**Supplementary Table 4. Sera of type 2 diabetes patients treated with a gliptin (T2D+g) or without gliptin medication (T2D) and healthy controls recognize BP180 epitopes differently in immunoblotting.** The immunoblotting data shown in Supplementary table 3 were analyzed pairwise with the Fisher-Freeman-Halton’s test for each fusion protein (FP) and full-length recombinant BP180 (BP180). Test values and 2‑sided P values are reported. Statistically significant P values (P < 0.05) and corresponding test values are highlighted in bold font.

|  |  | **FP1** | **FP2** | **FP3** | **FP4** | **FP5** | **FP6** | **FP7** | **FP8** | **FP9** | **FP10** | **FP11** | **FP12** | **FP13** | **BP180** |
| --- | --- | --- | --- | --- | --- | --- | --- | --- | --- | --- | --- | --- | --- | --- | --- |
| Ctrl vs  T2D | test value | **8.568** | 4.836 | **8.610** | **8.074** | - | **7.858** | **8.863** | 4.031 | 5.072 | - | - | - | - | **10.758** |
|  | P value | **0.017** | 0.127 | **0.026** | **0.035** | - | **0.034** | **0.008** | 0.193 | 0.139 | 1.000 | 0.492 | 0.238 | 0.104 | **0.004** |
| Ctrl vs  T2D+g | test value | **5.784** | 3.210 | 5.260 | 4.834 | - | 5.657 | 1.288 | 3.262 | 3.421 | 1.122 | - | - | - | 2.840 |
|  | P value | **0.029** | 0.417 | 0.150 | 0.191 | 1.000 | 0.103 | 0.804 | 0.413 | 0.193 | 1.000 | 0.488 | - | 0.452 | 0.242 |
| T2D vs  T2D+g | test value | 2.914 | 6.887 | 6.508 | 5.941 | - | 1.226 | **8.429** | 1.222 | 0.924 | 1.164 | - | - | - | **10.216** |
|  | P value | 0.416 | 0.057 | 0.070 | 0.084 | 0.486 | 0.845 | **0.008** | 0.733 | 1.000 | 0.795 | 1.000 | 0.229 | **0.019** | **0.007** |

# Supplementary Figures

**Supplementary Figure 1. Full-length BP180 ELISA cut-off value determined from received operator characteristic ROC curve and Youden index vs cut-off plot.** Sensitivity and 1 minus specificity and cut-off and Youden index from supplementary table 1 were plotted.


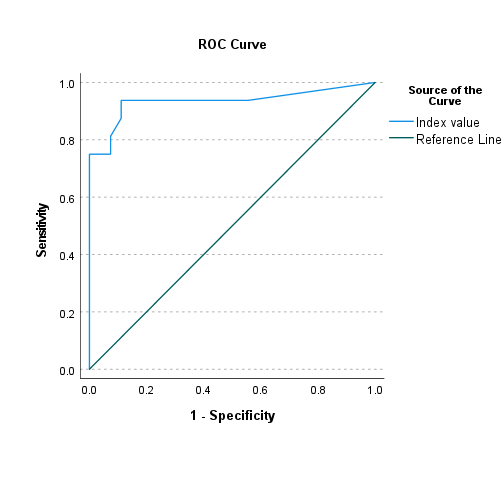

Supplement: Supplementary file 1 [file DataSheet_1.docx]
